# Unexpected selection to retain high GC content and splicing enhancers within exons of multiexonic lncRNA loci

**DOI:** 10.1261/rna.047324.114

**Published:** 2015-03

**Authors:** Wilfried Haerty, Chris P. Ponting

**Affiliations:** MRC Functional Genomics Unit, Department of Physiology, Anatomy and Genetics, University of Oxford, Oxford OX1 3PT, United Kingdom

**Keywords:** intergenic long noncoding RNAs, splicing, ESEs, GC content, selection

## Abstract

If sequencing was possible only for genomes, and not for RNAs or proteins, then functional protein-coding exons would be recognizable by their unusual patterns of nucleotide composition, specifically a high GC content across the body of exons, and an unusual nucleotide content near their edges. RNAs and proteins can, of course, be sequenced but the extent of functionality of intergenic long noncoding RNAs (lncRNAs) remains under question owing to their low nucleotide conservation. Inspired by the nucleotide composition patterns of protein-coding exons, we sought evidence for functionality across lncRNA loci from diverse species. We found that such patterns across multiexonic lncRNA loci mirror those of protein-coding genes, although to a lesser degree: Specifically, compared with introns, lncRNA exons are GC rich. Additionally we report evidence for the action of purifying selection to preserve exonic splicing enhancers within human multiexonic lncRNAs and nucleotide composition in fruit fly lncRNAs. Our findings provide evidence for selection for more efficient rates of transcription and splicing within lncRNA loci. Despite only a minor proportion of their RNA bases being constrained, multiexonic intergenic lncRNAs appear to require accurate splicing of their exons to transact their function.

## INTRODUCTION

Nucleotide composition has long been known to vary greatly among long genomic regions (Eyre-Walker and Hurst 2001; Duret and Galtier 2009). It also varies at shorter scales between coding regions and their flanking sequences (Louie et al. 2003), and shorter still within protein-coding genes between their exons and introns (Louie et al. 2003; Schwartz et al. 2009). The trend of higher GC content in exons over introns is a hallmark of coding sequences (Amit et al. 2012) and has been interpreted as implying more efficient transcription, splicing, or translation (Kudla et al. 2006; Amit et al. 2012). Nucleotide compositional variation across exons has been associated with short motifs proximal to exon–intron boundaries that either enhance or inhibit splicing (Mount et al. 1992; Fairbrother et al. 2002; Wang et al. 2004).

While these features are well known for protein-coding sequences, as are their molecular functions, much remains to be learned for the thousands of intergenic long (≥200 nt) noncoding RNAs (lncRNAs) that have been predicted to be transcribed from animal genomes (Ulitsky and Bartel 2013). Few such loci have been experimentally characterized, but those that have possess roles in dosage compensation in human and fruit fly (Kay et al. 1993; Kelley et al. 1999), splicing regulation, phosphorylation, chromatin remodeling, and pluripotency maintenance (for reviews, see Ulitsky and Bartel 2013; Ponting et al. 2009). Function, for some lncRNA loci, is conveyed by the act of transcription across the locus (transcriptional interference and/or chromatin remodeling) with the resulting transcript being functionally inert (Latos et al. 2012; Yoo et al. 2012), whereas for others it is mediated by the RNA transcript itself (for example: *Xist*, *Paupar*, Brockdorff et al. 1992; Vance et al. 2014). It remains unclear, however, how prevalent are these two classes of lncRNA mechanism. LncRNA loci are found in diverse genomic contexts (enhancer, promoter-associated, intergenic, intronic, antisense; for review, see Qureshi and Mehler 2012) and their transcripts, either polyadenylated or nonpolyadenylated, can be located in diverse cellular compartments (Derrien et al. 2012; van Heesch et al. 2014). They also vary widely in size, ranging from as few as 200 nt to >8 kb for loci such as *Malat1*. In addition lncRNA loci can either be composed of a single exon (for example, *Malat1*, *Paupar*) or of multiple exons (*Hotair* and *Xist*).

An improved understanding of the potential biological functions of intergenic lncRNA loci could derive from interrogating their nucleotide sequence and composition. Previous studies have attempted to identify functional domains within lncRNA by predicting RNA secondary structures or the potential interaction forces between lncRNA loci and protein-coding sequences (Bellucci et al. 2011). RNA secondary structure predictions have tentatively ascribed functional regions to lncRNAs (Smith et al. 2013) but these have tended to suffer from high false-positive rates (Babak et al. 2007). In general, while lncRNA exons are better conserved than their introns, most strikingly so for *Drosophila* sequences, their conservation is exceedingly modest in human, whose contrast to fruit flies is a presumed result of humans’ much lower effective population size (Haerty and Ponting 2013).

We sought to further understand the degree by which intergenic lncRNAs contribute to biological function in diverse animal species. We looked beyond the known modest sequence conservation in lncRNA exons by examining their nucleotide composition, and their hallmarks of active transcription, splicing, and evolution, specifically in comparison with protein-coding genes. Unexpectedly, we find that selection acts on exonic splicing enhancers in human and on nucleotide substitutions in lncRNAs in fruit fly.

Our findings of signatures of efficient splicing and selection, similar to those evident within protein-coding genes, indicate that despite their exons’ low degrees of sequence conservation, many multiexonic lncRNA loci are likely to possess spliced RNA-dependent functions.

## RESULTS

We focused our studies on 66,500 intergenic lncRNAs from fruit fly, zebrafish, coelacanth, mouse, and human, respectively (Table 1). This wide phyletic range allowed us to obtain a broad perspective of lncRNA evolution and function across metazoan evolution. In previous studies on nematode (Nam and Bartel 2012), zebrafish, and mouse (Ulitsky et al. 2011), intergenic lncRNA loci were found to exhibit a GC content that is higher than their flanking intergenic region yet that is significantly lower than that for protein-coding sequences. Exonic and intronic GC contents and splicing motifs were, however, not compared in these previous studies.

**TABLE 1.**
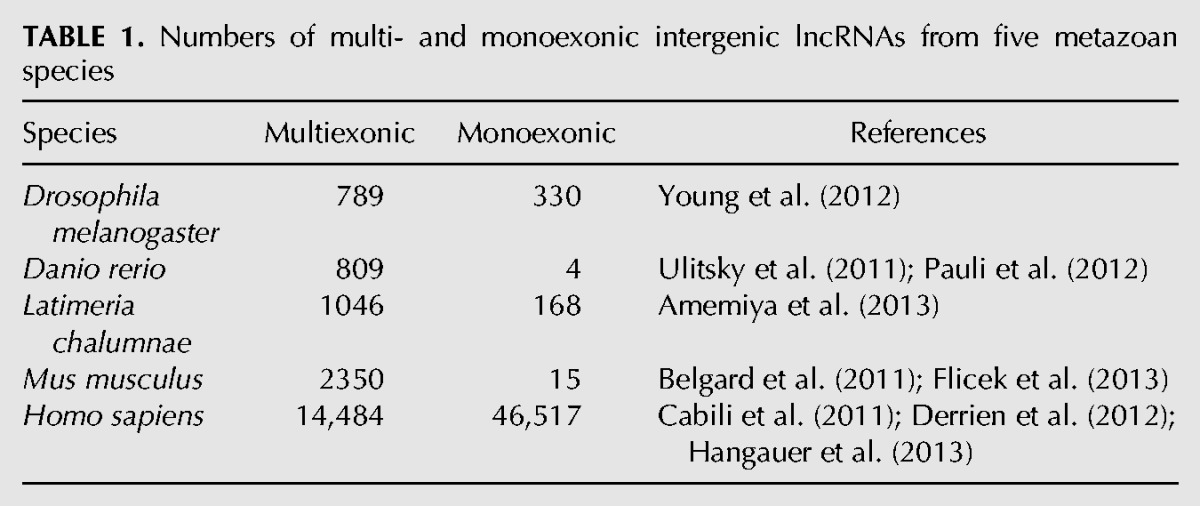
Numbers of multi- and monoexonic intergenic lncRNAs from five metazoan species

### Elevated GC content in lncRNA exons over introns for diverse metazoans

**FIGURE 1.**
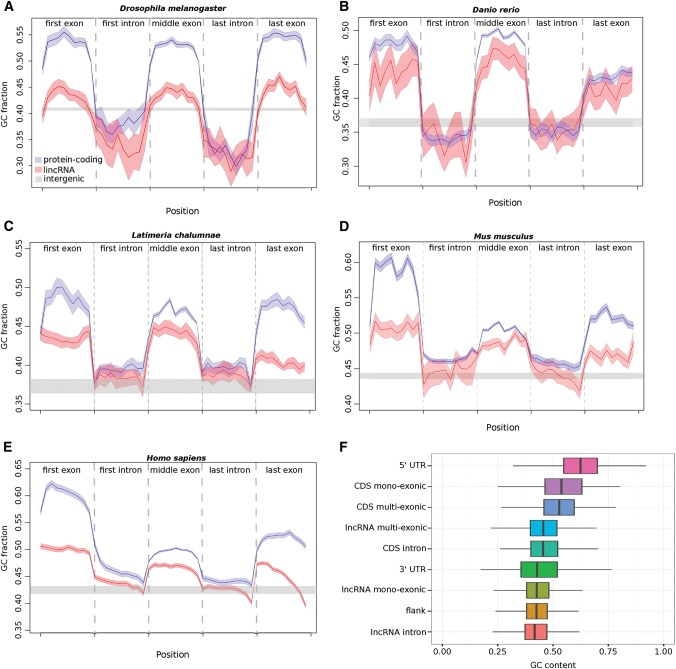
GC content variation across multiexonic protein-coding (blue) and intergenic lncRNA loci (red) in (*A*) *Drosophila melanogaster* (Young et al. 2012), (*B*) *Danio rerio* (Ulitsky et al. 2011; Pauli et al. 2012), (*C*) *Latimeria chalumnae* (Amemiya et al. 2013), (*D*) *Mus musculus* (Belgard et al. 2011), and (*E*) *Homo sapiens* (Cabili et al. 2011; Derrien et al. 2012). Nonoverlapping windows each sampling 10% of the sequences were used. The gray band indicates GC content of flanking intergenic sequences. (*F*) Comparison of exonic GC content between multiexonic and monoexonic intergenic lncRNA loci as well as 5′- and 3′-UTR exons from protein-coding genes in human.

Exons of intergenic multiexonic lncRNAs for all five species that we considered contain significantly higher GC content than either their intronic or flanking intergenic sequences; this pattern of GC content variation across the gene model mirrors that seen for their neighboring protein-coding gene models, albeit to a reduced extent (Fig. 1A–E). Single exon intergenic lncRNAs exhibit lower GC content that is only marginally greater than that of their untranscribed flanking sequences, and thus significantly lower than for multiexonic intergenic lncRNAs (*P* < 0.001) (Fig. 1F). The nucleotide composition of exons from multiexonic lncRNA loci is between that for 5′ or 3′ UTRs (*P* < 0.05 in both comparisons), while monoexonic loci have the lowest GC content of all exonic categories we considered (*P* < 0.05) (Fig. 1F) with the exception of 3′-UTR exons (*P* > 0.05). In their elevated GC content, therefore, multiexonic lncRNAs are more similar to protein-coding loci than they are to monoexonic lncRNAs.

The elevated GC content seen for both multiexonic lncRNA and protein-coding gene exons might indicate that predicted lncRNA exons are, instead, protein-coding. Indeed, a number of proposed lncRNA loci, at least in *Drosophila* (Ladoukakis et al. 2011), zebrafish, and human (Bazzini et al. 2014), may encode small open-reading frames (smORFs <100 amino acids). Because of the high GC content of protein-coding exons, we compared the nucleotide content of these smORFs to the remaining intergenic lnRNAs. Noncoding multiexonic lncRNAs exhibit GC levels that are higher than for introns yet that are significantly lower than for these multiexonic smORFs (Mann–Whitney test, *P* = 0.032, Supplemental Fig. 1) arguing that the two locus classes are distinct. In all subsequent analyses all smORF transcripts were excluded from consideration.

Another potential factor influencing the nucleotide composition of lncRNAs could be the embedding of DNA functional elements within them. Indeed, nucleotide composition and GC content more specifically have been directly associated with human transcription factor binding sites (TFBS) (White et al. 2013). We thus considered associations between lncRNA exonic GC content and the presence of TFBS or enhancer elements defined by the FANTOM5 consortium (Andersson et al. 2014) within these exons. Within both monoexonic and multiexonic lncRNAs we observed a significant association between nucleotide composition and the occurrence of TFBS or enhancers (*P* < 0.001). Even within the same locus, exons with a transcription factor binding site have a significantly higher GC content than exons without such sites (Mann–Whitney test *P* < 2.2 × 10^−16^, Supplemental Fig. 2). However, multiexonic lncRNAs remain GC rich compared with monoexonic loci even when they do not contain experimental TFBS or enhancers (Mann–Whitney test *P* < 2.2 × 10^−16^, Supplemental Fig. 3).

### GC content across multiexonic gene models

Protein-coding exons exhibit particularly elevated exonic GC content and strong splice sites when they are flanked by long introns (Amit et al. 2012). We found, similarly, that the elevated GC content of lncRNA exons over their flanking introns is significantly greater for long (third quartile) introns than for shorter (first quartile) introns (Mann–Whitney tests, *P* = 1.4 × 10^−12^ and *P* = 3.4 × 10^−6^ for 5′ and 3′ introns, respectively, Supplemental Fig. 4). This exon–intron differential GC content is significantly correlated with the length of the flanking introns (ρ = 0.085, *P* = 9.9 × 10^−14^ and ρ = 0.057, *P* = 6.6 × 10^−7^ for 5′ and 3′ introns, respectively). If multiexonic lncRNA loci are commonly functionally spliced then it might be expected that splice sites would be significantly stronger (as inferred from their information content) (Yeo and Burge 2004) when flanked by long introns. This, indeed, was found to be the case (Mann–Whitney test, *P* = 2.8 × 10^−6^ and *P* = 2.1 × 10^−5^ for the 5′ and 3′ splice sites, respectively, comparing the 25% shortest or longest introns). Additionally, we observed a significant positive correlation between intron size and splice site strength in both coding (ρ = 0.081, *P* < 2.2 × 10^−16^, and ρ = 0.076, *P* < 2.2 × 10^−16^ for 5′ and 3′ splice sites, respectively) and lncRNA (ρ = 0.087, *P* = 2.2 × 10^−14^, and ρ = 0.081, *P* = 1.2 × 10^−14^ for 5′ and 3′ splice sites, respectively) loci.

**FIGURE 2.**
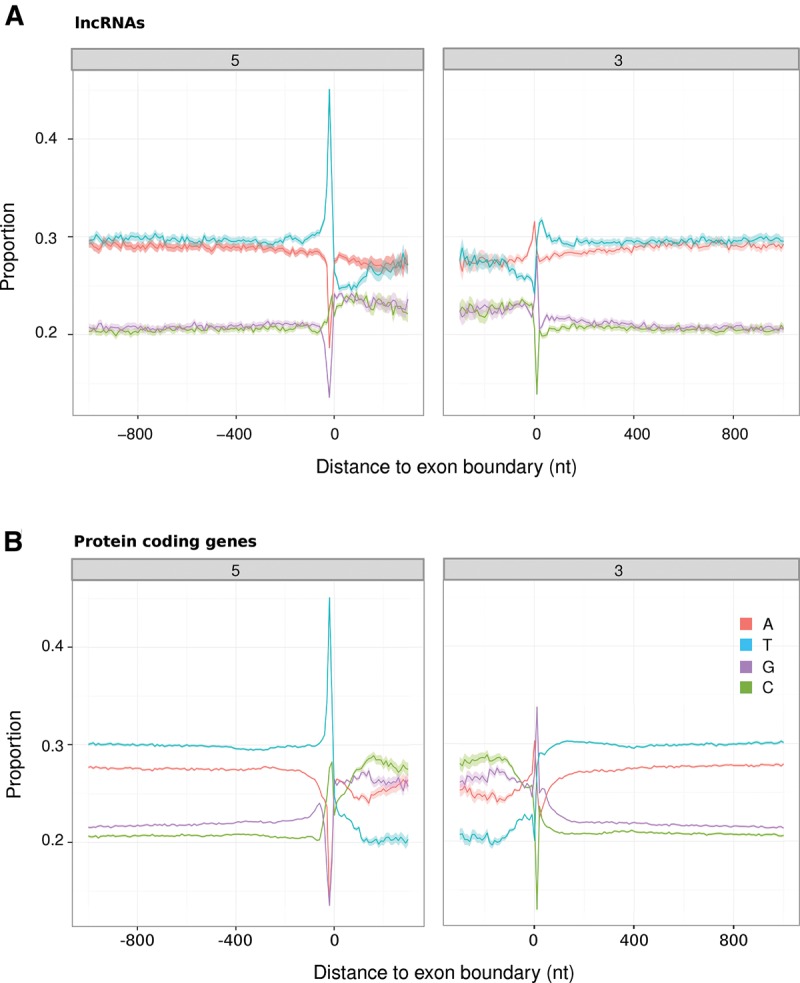
Comparison of nucleotide composition at the exon–intron boundaries of internal exons within human intergenic lncRNAs (*A*) and protein-coding genes (*B*). The areas designate the fifth and 95th percent confidence intervals.

**FIGURE 3.**
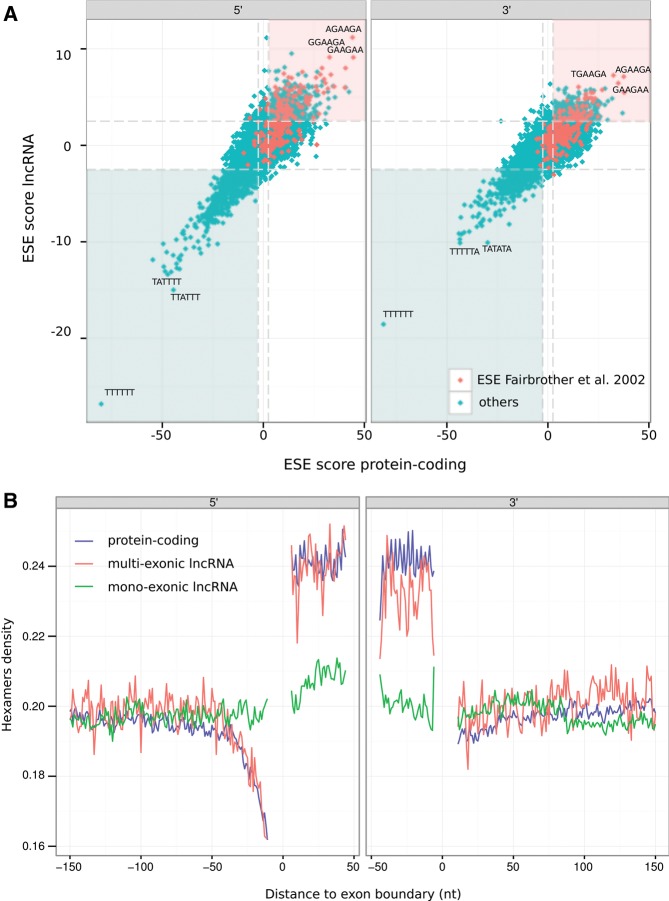
(*A*) Comparison of hexamer scores (as per Fairbrother et al. 2002) between human protein-coding and lncRNA exons. Red dots represent the ESEs previously identified by Fairbrother et al. (2002). Red and blue areas contain motifs that are enriched within exons or within introns, respectively, of both protein-coding and lncRNAs (ESEscore > |2.5|, Fairbrother et al. 2002). (*B*) Proportion of hexamers that are ESEs (Fairbrother et al. 2002; Zhang and Chasin 2004; Goren et al. 2006) at the 5′ and 3′ exon–intron boundaries within human protein-coding (blue), multiexonic lncRNAs (red), and monoexonic lncRNA (green) loci.

We next examined sequence adjacent to lncRNA exon–intron boundaries and focused on 6136 human intergenic lncRNAs with at least three exons and constitutively spliced introns. Although not as dramatic as for the protein-coding exons, the compositional bias of their internal exons is due to increases of both G and C nucleotides equally. Less expected was the strong T enrichment within 40 nt upstream of the acceptor splice site signal which indicates the presence of the polypyrimidine tract as well as the depletion in T (but not A) toward both 5′ and 3′ exonic boundaries (Fig. 2). To consider whether this depletion reflected the preferential location of purine-rich exonic splicing enhancers (ESEs) toward exon–intron boundaries we predicted hexamers that are enriched near (<50 nt) to these boundaries. These lncRNA exon hexamers were found to be highly concordant with the set of ESE motifs previously identified by Fairbrother et al. (2002) for protein-coding exons (3.80- and 3.87-fold enrichment, respectively, for the 5′ and 3′ exonic boundaries if Δ*E* ≥ 2.5). The ESE scores (calculated as per Fairbrother et al. 2002) were also highly correlated between protein-coding and lncRNA sequences (Spearman's rank correlation coefficient, ρ = 0.75 and ρ = 0.76 for the exonic 5′ and 3′ ends, respectively) (Fig. 3A).

### Evolutionary conservation of ESESs in multiexonic lncRNAs

**FIGURE 4.**
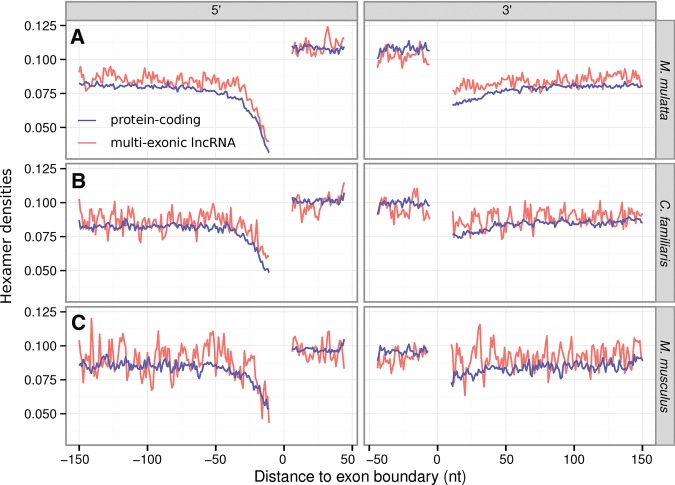
Proportion of hexamers that are predicted exonic splicing enhancers (Fairbrother et al. 2002; Zhang and Chasin 2004; Goren et al. 2006) within (*A*) *Macaca mulata*, (*B*) *Canis familiaris*, (*C*) *Mus musculus* genomic sequences that align to predicted exonic splicing enhancers in human intergenic lncRNA exons.

Conservation of human lncRNA transcription across mammalian evolution is the exception rather than the rule, which contrasts with the high evolutionary conservation of protein-coding mRNA transcription (Kutter et al. 2012; Necsulea et al. 2014). It was unexpected, therefore, that exonic predicted ESE levels were equivalent to those of highly conserved protein-coding genes for macaque, dog or mouse genomic sequence orthologous to predicted ESEs in human multiexonic lncRNAs (Fig. 4). We conclude, at least for 171 human multiexonic intergenic lncRNAs whose ESEs could be aligned to genomic sequence of all these other mammals, that the density of predicted ESEs was preserved across 100 million years of mammalian evolution, as they are for protein-coding genes.

Using multiple species’ genome alignments, we investigated the pattern of nucleotide conservation associated with exon splicing. We found that nucleotide conservation in lncRNA exons across primates is greatest near to the exon boundaries in comparison to sequences of the same length from the middle of the same exons (5′: *P* = 6.6 × 10^−16^ and 3′: *P* = 7.1 × 10^−10^ after Bonferroni correction; Materials and Methods). Additionally, we observed sequence adjacent to the splice acceptor site at the 5′ ends of lncRNA exons significantly better conserved than sequence at 3′ ends (Kruskal–Wallis test, *P* = 1.0 × 10^−5^). This finding, which was previously seen for protein-coding sequences (Chamary and Hurst 2005; Chamary et al. 2006; Parmley et al. 2007; Warnecke and Hurst 2007), reflects the sequence requirements for the spliced RNA-dependent functions of multiexonic lncRNAs.

**FIGURE 5.**
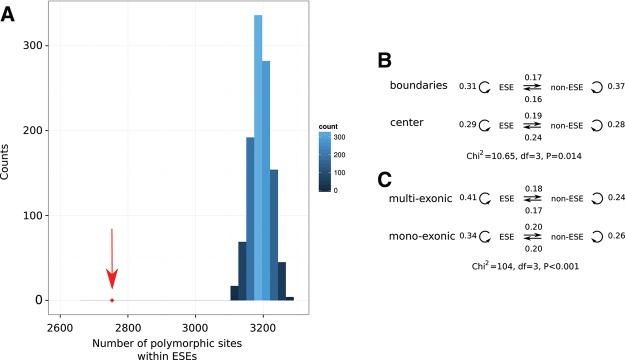
(*A*) Comparison of the observed (2743, red arrow) and the number of polymorphic sites within ESE sequences (predicted as per Fairbrother et al. 2002; Zhang and Chasin 2004; Goren et al. 2006) expected by simulation within human intergenic multiexonic lncRNAs. The distribution of expected number of polymorphic sites within ESEs is based on 1000 randomizations of SNPs within multiexonic lncRNA exons accounting for base composition. (*B*) Comparison of the nucleotide substitution patterns with respect to ESE motifs within 20 nt of exon boundaries and 20 nt located at the center of lncRNA exons. (*C*) Comparison of the nucleotide substitution patterns with respect to ESE motifs identified within multiexonic and monoexonic lncRNAs.

To investigate whether purifying selection has acted on human multiexonic lncRNA ESE sequences we compared derived allele frequency distributions (Vitti et al. 2013) between single nucleotide variants (SNV) occurring within ESE or non-ESE motifs within 50 nt of exon boundaries. No differences were found, perhaps owing to the method being underpowered to detect selection from such small sequence samples. Nonetheless, the density of human polymorphic sites within these multiexonic lncRNA predicted ESEs was significantly lower (empirical *P* < 0.001) (Fig. 5A; Supplemental Fig. 5) relative to composition matched random samples (see Materials and Methods), and substitutions at these sites, when comparing human sequence with either chimpanzee or macaque, are significantly depleted (empirical *P* < 0.001 and *P* < 0.001, respectively).

Next we extended the analysis to consider whether nucleotide substitutions conserve ESE (or non-ESE) status. We observed significant differences in the nucleotide substitution patterns characterized by a depletion of ESE creating and ESE disrupting mutations within lncRNA exon boundaries relative to the center of exons (χ^2^, *P* = 0.014) (Fig. 5B). Importantly, this nucleotide substitution pattern mirrors that previously reported by Fairbrother et al. (2004a) for protein-coding ESEs. A similar analysis contrasting nucleotide substitution patterns between multiexonic and monoexonic lncRNA loci shows mutations preserving the ESEs and fewer ESE creating and ESE disrupting mutations within multiexonic lncRNA (Fig. 5C).

The identification of increased density and nucleotide conservation of ESEs at orthologous loci in mammalian species, as well as mutation and substitution biases affecting discrete sequences, indicate that even though lncRNA loci are rapidly evolving as a whole, constraint on short nucleotide sequences has acted, over relatively recent human evolution, on ensuring efficient splicing of these multiexonic lncRNAs.

### Multiexonic lncRNA loci share with protein-coding genes several genomic and transcriptional features

The unexpected splicing and GC content similarities between human multiexonic lncRNAs and protein-coding genes motivated us to consider additional features, specifically those that have been attributed to transcriptional regulation. Protein-coding exons exhibit an unusually high level of binding to nucleosomes, perhaps because high GC-content DNA structure is less rigid, and they also appear to slow the procession of RNA polymerase II, perhaps to assist in the proper recognition of regulatory elements by the splicing machinery (Schwartz et al. 2009; Gelfman and Ast 2013). Coding exons tend also to have higher fractions of CpG dinucleotides that are methylated than introns (Gelfman et al. 2013), and higher densities of trimethylated histone 3 lysine 36 (H3K36me3) marks (Schwartz et al. 2009; Luco et al. 2010).

**FIGURE 6.**
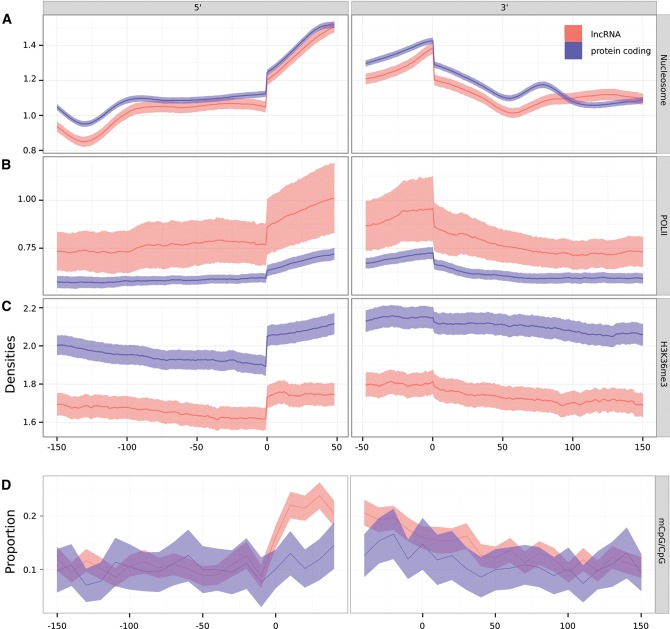
Comparison of (*A*) nucleosome, (*B*) RNA polymerase II (POLII), and (*C*) H3K36me3 reads densities, and (*D*) the proportion of methylated CpG (mCpG) over the number of CpG at the 5′ and 3′ boundaries of intermediate protein-coding (blue) and intergenic lncRNA (red) exons.

Using data for the human lymphoblastoid cell line Gm12878 (ENCODE Project Consortium et al. 2012), we found that lncRNA exons exhibit elevations in nucleosome binding, and RNA polymerase II read densities relative to their introns (Fig. 6A,B). In contrast, the density of H3K36me3 marks, is only marginally higher for multiexonic lncRNA exons than introns (Fig. 6C). In comparison to a set of protein-coding genes selected to match the expression levels of lncRNAs, we observed comparable nucleosome binding at the lncRNA exon–intron boundary, and lower density of H3K36me3 marks. Surprisingly, we found the density of Pol II reads to be higher for lncRNA loci than expression level matched protein-coding sequences. This increased density could reflect greater polymerase pausing at the lncRNA exons relative to protein-coding exons or highlight the greater instability of lncRNA transcripts (Clark et al. 2012). In contrast to protein-coding exons, we did not observe an increased proportion of methylated CpG within lncRNA exon boundaries. This is likely the consequence of the low number of lncRNA loci identified in this data set (604) leading a reduced power to detect such events, because a significant enrichment is observed when using the full lncRNA annotation regardless of the expression calls in H1 cells (Supplemental Fig. 6; Lister et al. 2009).

In summary, multiexonic lncRNA loci are associated with signatures of enhanced splicing efficiency, some of which are similar to those previously observed in protein-coding genes, which indicates their transcripts’ functionality.

### Evolution of intergenic lncRNA exon nucleotide composition

We then sought to analyze the evolutionary forces shaping the nucleotide composition of intergenic multiexonic lncRNAs. To do so we analyzed both the mutation and substitution patterns (A,T → G,C and G,C → A,T) at different genomic features. If base composition is at equilibrium, we expect sequences to have similar ratios of polymorphism to divergence for the different classes. Any departure would be indicative of action of nonselective (biased gene conversion) or selective forces acting on the nucleotide composition (Haddrill and Charlesworth 2008). Our analysis focused on *Drosophila* (Mackay et al. 2012), a species chosen owing to its larger effective population size and therefore greater efficiency of selection than human (Eyre-Walker et al. 2002; Li and Durbin 2011).

**FIGURE 7.**
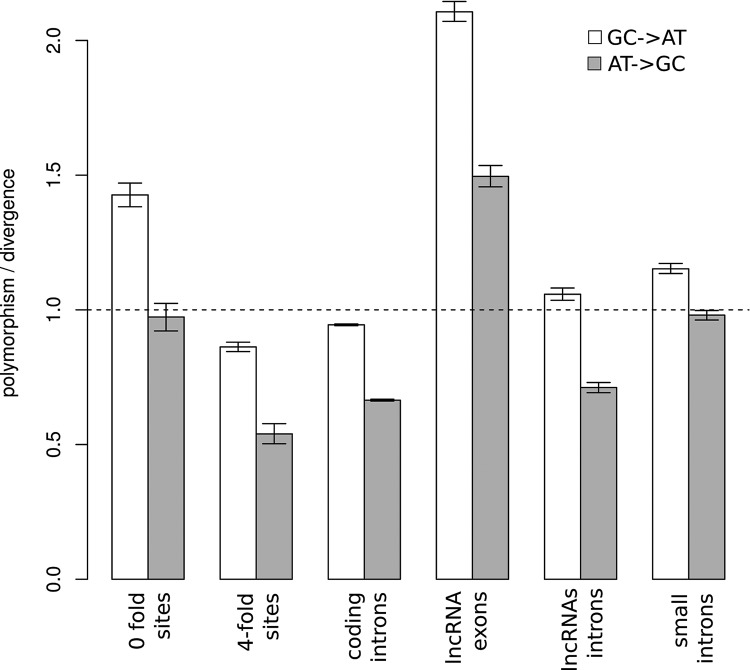
Comparison of polymorphism to divergence ratio depending upon mutation bias (AT → GC, GC → AT) between different genomic categories. Small introns include introns from protein-coding genes that are <86 nt.

As expected from previous studies in *D. melanogaster* we observed a general excess of GC → AT relative to AT → GC that is indicative of the AT biased mutation rate in this species (DuMont et al. 2009; Singh et al. 2009). Most importantly we observed strong differences between nondegenerate and lncRNA exonic sites relative to intronic sites (Fig. 7). LncRNA exons and nondegenerate sites are characterized by a significant excess of polymorphism over divergence (χ^2^ test, *P* < 0.001) (Fig. 7) which indicates the action of purifying selection (Haerty and Ponting 2013). Furthermore for these sites we found the ratio polymorphism_GC → AT_/divergenc_GC → AT_ to be significantly greater than polymorphism_AT → GC_/divergenc_AT → GC_. These results could be interpreted as the consequence of purifying selection acting on nucleotide composition that disfavors the fixation of G,C to A,T mutations within multiexonic lncRNA exons. High GC content thus tends to confer functional benefit on spliced lncRNA exons in fruit flies.

Finally, we sought to provide evidence for the reasons underlying the elevated GC content of human multiexonic lncRNA exons. After taking into account, using partial correlation analysis, the potential association among multiple factors (recombination rate, intron size, number of exons, expression level) (Supplemental Fig. 7), GC content remained weakly but significantly positively correlated with multiexonic lncRNA expression levels, specifically their maximum expression values across 16 tissues (*P* < 0.001) or their tissue expression breadth (*P* < 0.001). High GC sequence in protein-coding exons has been associated with greater transcriptional activity (Kudla et al. 2006). Our results thus imply that human multiexonic lncRNAs exhibit protein-coding gene-like signatures of selection for enhanced transcriptional and splicing efficiencies.

## DISCUSSION

Human multiexonic lncRNA loci are very modestly constrained in their exons, relative to their introns, which we have interpreted as implying either that their functions contribute little to organismal fitness or that their functionality is conveyed by only a small minority of their sequences (Ponjavic and Ponting 2007; Haerty and Ponting 2013). The first of these two possibilities is further suggested by the frequent lack of conserved transcription of orthologous lncRNAs across diverse mammals (Kutter et al. 2012; Necsulea et al. 2014). However, the latter possibility is supported by our observations that, akin to protein-coding genes, (i) evolutionary constraint is more concentrated near to human spliced lncRNA intron–exon boundaries, (ii) such regions contain an unusually high density of ESEs, and (iii) these ESEs are unexpectedly preserved in orthologous sequence in sequence-divergent mammals. Consequently, in contrast to a previous study which indicated that transcription of predominantly monoexonic lncRNAs is not conserved in a single tissue, adult liver, across eutherian mammals (Kutter et al. 2012), our findings indicate that transcription and efficient splicing of multiexonic lncRNAs will often be conserved across mammals, perhaps in spatiotemporally distinct tissues.

### Intergenic lncRNA exons are GC rich compared with introns or monoexonic intergenic lncRNAs

Elevated GC content within exonic sequences has long been a hallmark of protein-coding sequences. Unexpectedly, we showed that this signature is also shared by intergenic multiexonic lncRNAs across five eukaryotic species although to a lower extent than the variation observed between protein-coding exons and introns.

However, multiple neutral processes could also explain the observed elevated GC content within lncRNA exons and in many cases their effects have been wrongly ascribed to the action of selection. The most important mechanism involves GC-biased gene conversion, a process by which, during recombination, the mismatch repair mechanism favors the propagation of G,C over A,T alleles. GC-biased gene conversion within lncRNA loci would be consistent with the positive correlation of genomic GC content and recombination rate (Duret and Galtier 2009) as we observed for lncRNA loci. Nevertheless, because GC-biased gene conversion does not differentiate between exons and introns, it alone cannot explain their different GC contents. For the same reason, transcription-coupled repair (Polak et al. 2010), whose effects on nucleotide content extend across complete transcribed loci, also cannot explain the variation in GC content between exons and introns.

Higher GC content within protein-coding exons has been proposed to be a consequence of selection on the efficiency or robustness of translation (Akashi 1995; Drummond and Wilke 2009; Tuller et al. 2010). The vast majority of the lncRNA loci that we analyzed are unlikely to be translated (Guttman et al. 2013; Wilhelm et al. 2014), hence their high GC exonic content cannot be explained in this manner. Increased lncRNA exon GC content might also be ascribed to selection for the formation and the maintenance of GC-rich RNA secondary structures. Previous analyses reported enrichment for conserved secondary structures within lncRNA (Smith et al. 2013) and a correlation between folding propensity and lncRNA expression (Managadze et al. 2011). The major difficulty still resides in the high false discovery rate associated with de novo predictions. However, the experimental identification of regions involved in secondary structures either using Parallel Analysis of RNA Structures (PARS), Parallel Analysis of RNA structures with Temperature Elevation (PARTE), or ds/ssRNA-seq (for review, see Mortimer et al. 2014) should overcome this issue in the future.

Finally, the elevated GC content within exonic sequences could also reflect that efficient transcription of these loci is under selection across multiple species. The positive correlation we found between nucleotide composition and expression levels of human intergenic lncRNAs is consistent with this hypothesis. Previously Kudla et al. (2006) showed that manipulation of the GC3 content of a sequence dramatically affects its transcription and translation rates in human cells.

In comparison to multiexonic lncRNAs, we failed to observe elevated GC content in large numbers of proposed monoexonic lncRNAs, for example the 45,905 (89.2%) that have single exons in the set of Hangauer et al. (2013). These models also show significantly weaker biases in nucleosome binding, polymerase occupancy or H3K36me3 occupancy relative to multiexonic loci. Consequently, we conclude either that the GC content profile of multiexonic lncRNAs can be explained solely by their splicing requirements or that sets of monoexonic lncRNAs contain false-positive predictions that greatly outnumber well-described monoexonic lncRNA such as *Malat1*, *Neat1*, and *Paupar* that have demonstrated cellular functions (Clemson et al. 2009; Bernard et al. 2010; Tripathi et al. 2010; Vance et al. 2014). Monoexonic loci can be wrongly predicted because of genomic DNA contamination in RNA sequencing libraries, or serendipitous transcription. It is also possible that such predictions represent RNAs with functions that are very different from multiexonic lncRNAs, such as those derived from transcription across active genomic regions such as enhancers (Marques et al. 2013).

### Splicing-associated purifying selection in multiexonic lncRNAs

The mechanism of exon splicing within protein-coding transcripts has long been associated with specific chromatin marks (Schwartz et al. 2009; Luco et al. 2011) but most importantly with purifying selection on discrete exonic and intronic sequences within protein-coding genes in eukaryotes (Chamary et al. 2006; Warnecke and Hurst 2007). It is therefore not fully unexpected that we found enrichments at the lncRNA exon–intron boundaries of factors or features that previously have been associated with “optimal” splice site choice (Pol II, or H3K36me3 densities, Schwartz et al. 2009; Luco et al. 2010; Gelfman and Ast 2013).

We previously reported no significant evidence for purifying selection acting on human intergenic lncRNAs when studying full loci (Haerty and Ponting 2013). However, an absence of evidence for selection at the full gene model level does not preclude short functional regions selectively constrained. Indeed, lncRNA exonic sequences predicted to encode splicing regulatory elements show increased conservation and significant substitution biases against ESE disruption and ESE creation. Remarkably, the same patterns of elevated nucleotide conservation and substitution biases are well known for protein-coding sequences. For instance, within human populations, Fairbrother et al. (2004a) identified purifying selection acting on ESEs. The authors reported lower ESE disruption and ESE creation than expected, highlighting the effect of natural selection on these regulatory motifs. Additionally, similar to our conclusions of a greater conservation of ESEs close to lncRNA exon boundaries, the authors also showed that the selective pressure on ESEs was also stronger close to splice sites. Several subsequent analyses of ESEs within protein-coding sequences reached similar conclusions either using multispecies comparisons or larger SNV data sets in human (Parmley and Hurst 2007; Ke et al. 2008; Cáceres and Hurst 2013).

Overall, and following on previous analyses of splicing in protein-coding genes (Fairbrother et al. 2004b; Schwartz et al. 2009; Amit et al. 2012; Gelfman and Ast 2013) and despite low selective constraints detected at the full locus level (Ponjavic et al. 2007; Marques and Ponting 2009; Ulitsky and Bartel 2013), we have identified strong indications that splicing of multiexonic lncRNA loci is likely to often be required for their function. These include (1) a significantly increased level of nucleotide conservation across primates for human lncRNA exonic sequence near to their intron boundaries relative to their internal regions (see Haerty and Ponting 2014) and (2) a significant depletion of polymorphic sites within predicted ESE motifs. These observations are surprising because intergenic lncRNA sequence tend to be very poorly conserved, and evidence of selection at the whole locus level is at the best weak (Marques and Ponting 2009; Haerty and Ponting 2013).

Our results indicate that these lncRNA loci predominantly possess spliced RNA sequence-dependent functions that are conveyed by only minor proportions (<5%) of their exonic sequence (Ponjavic et al. 2007); moreover, as for protein-coding loci, their regulatory sequence and composition appear to have been under selection for efficient transcription and splicing. Together, our observations imply that multiexonic lncRNA loci often convey spliced RNA-dependent functions that are widely conserved among mammals. Our findings cannot, however, distinguish between RNA exon sequence-dependent functions that act locally, near to their sites of synthesis, and those that act more distally, such as chromatin guides and scaffolds (Kung et al. 2013) or competitive endogenous lncRNAs (Marques et al. 2011).

Our observations thus better discriminate functional lncRNAs and identify functional elements, namely splice sites, which can now be targeted for disruption, for example, using CRISPR/Cas9 technology in high-throughput phenotypic assays.

## MATERIALS AND METHODS

To allow comparisons with protein-coding sequences, whose compositional features are well-established, we focused our analyses on intergenic lncRNAs. Antisense, overlapping, and intronic lncRNAs were all discarded from published lncRNA data sets prior to analyses.

### Long intergenic noncoding RNA data sets

Intergenic lncRNAs were acquired from published sets (Table 1) for the fruit fly (*Drosophila melanogaster*), zebrafish (*Danio rerio*), coelacanth (*Latimeria chalumnae*), mouse (*Mus musculus*) and human (*Homo sapiens*). We considered a nonredundant, nonoverlapping set of human intergenic lncRNAs identified by Cabili et al. (2011), the ENCODE consortium (Derrien et al. 2012) or Hangauer et al. (2013) resulting in a total of 61,001 intergenic lncRNAs. We note that the Hangauer et al. data set is comprised of 45,905 (89.2%) single exon lncRNA models.

In order to avoid contamination in lncRNA sets derived from protein-coding sequences either through gene duplication or pseudogenization that might inflate their computed nucleotide content, we removed, prior to analyses, any locus that shared weak or strong sequence similarity with annotated protein sequences (as detected using BLASTN (*E* < 0.1) or whose genomic loci overlapped (in multigenome alignments, UCSC genome database http://genome.ucsc.edu/) with annotated protein-coding loci in other vertebrates species.

### Nucleotide sequence composition

All transcripts in each lncRNA locus were collapsed in order to derive their maximal extended exons. Exons (or introns) of lncRNA loci or their closest genomically neighboring protein-coding genes were partitioned into their first, middle, or last or their sole exons (single exon gene models) and their G + C proportions computed within windows that each represents a 10% portion (decile) of exonic sequence. This procedure was repeated for introns of multiexonic lncRNAs. It was also repeated for protein-coding genes, but only for those genes lying in genomic sequence adjacent to intergenic lncRNA loci, in order to match for nucleotide composition which is known to vary on the 100 kb–1 Mb scale. We also ran the same analysis on the flanking intergenic sequences after masking 500 nt adjacent to annotations. Our analyses made use of the hg19, mm10, danRer7, latCha1, and dm3 versions of genome assemblies, all acquired from the UCSC genome database.

### Exonic splicing regulatory elements

We implemented the RESCUE-ESE algorithm developed by Fairbrother et al. (2002, 2004b) to identify hexamers that are significantly enriched or depleted within exonic sequences relative to their flanking intronic sequences. We focused our analysis on all internal (“middle”) lncRNA exons that are longer than 100 nt and flanked on both sides by introns including at least 400 nt. Following Fairbrother et al. (2002) and Yeo et al. (2004), prior to analysis we masked nucleotides flanking the splice sites (5 nt for the 5′ exonic, intronic [donor], and 3′ exonic sequences and 20 nt for the 3′ intron [acceptor site]).

We took advantage of sets of 607 exonic splicing enhancer hexamers (ESEs) previously predicted by Fairbrother et al. (2002), Zhang and Chasin (2004) or Goren et al. (2006) to identify the density of predicted splicing regulatory elements in exonic sequence lying adjacent to exon–intron boundaries for internal exons within human lncRNA loci or protein-coding genes. Fifty nucleotides of exonic sequence and 150 nt of intronic sequence were considered: The first five exonic nucleotide and 10 intronic nucleotides were discarded from our analyses because of the string composition bias associated with these splice sites. In order to assess the rate of cross-species conservation of these regulatory sequences we used UCSC liftOver files (obtained from http://genome.ucsc.edu/) to project the human lncRNA sequence onto orthologous macaque (rheMac3 assembly), dog (canFam3), and mouse (mm10) genomes and calculated the density of predicted ESE motifs identified within these orthologous sequences.

### Epigenetic marks associated with splicing

Instead of considering predictions of nucleosome occupancy (Kaplan et al. 2009), which depend on nucleotide composition, we took advantage of experimental evidence for nucleosome binding (ENCODE Project Consortium et al. 2012).

Multiple factors have been proposed to regulate the splicing of exons in mammals (Brown et al. 2012). We used the signal tracks generated by the ENCODE consortium (ENCODE Project Consortium et al. 2012) to quantify the enrichment or depletion of nucleosome location, RNA polymerase II reads and H3K36me3 marks at multiexonic lncRNA exon–intron boundaries. These data were acquired in the Gm12878 cell line and only a subset was considered, namely for those loci that had uniquely mapped reads from RNA-seq for this cell line. For comparison, we performed the same analyses for all protein-coding genes expressed in either of these two cell lines. The difference in occupancy or enrichment between exonic and intronic sequences was tested using a Mann–Whitney test, focusing on the 50 exonic and 50 intronic nucleotides flanking each splice site. Because of the large difference in expression levels between protein-coding and lncRNA genes, we selected a subset of 2642 protein-coding sequences whose gene expression distribution in Gm12878 matches the expression distribution of lncRNA loci.

The proportion of methylated CpG dinucleotides near to lncRNA exon–intron boundaries was computed for each exonic position using data generated by Lister et al. (2009) (http://neomorph.salk.edu/human_methylome/data.html). As before, the protein-coding genes were sampled to match the expression of the lncRNAs in the H1 cell line (Lister et al. 2009).

### lncRNA composition and constraint

Factors such as recombination rate, gene structure, or gene expression level have previously been shown to correlate with protein-coding nucleotide composition (Chamary et al. 2006). Consequently, we used the corpcor partial correlations package from R (http://strimmerlab.org/software/corpcor/) to test, using a partial correlations approach, for associations between these genomic factors and GC content within lncRNA loci in fruit fly and/or human genomes. Significance of these partial correlations was assessed through randomization of values while keeping one parameter constant. For computation of partial correlations, we collected for sequence intervals their (i) recombination rate values (Fiston-Lavier et al. 2010; Kong et al. 2010), (ii) GC content for exons and introns, (iii) numbers of introns, (iv) expression level from 16 organs as part of the Human Body Map, and (v) the expression breadth across tissues (τ) (Larracuente et al. 2008).

To assess the distribution of polymorphic sites within lncRNA exons with respect to splicing regulatory elements we used polymorphism data from the 1000 Genomes Consortium (1000 Genomes Project Consortium et al. 2012). The SNV density within exonic spliced enhancers (ESEs, 607 motifs, Fairbrother et al. 2002; Zhang and Chasin 2004; Goren et al. 2006) was then compared with the expected SNV density based on 1000 random samples from the same sequences taking into account nucleotide composition. In order to also account for mutation biases associated with dinucleotide composition, the resampling analysis was performed again conserving the dinucleotide composition of the sequences.

Additionally, using the pairwise alignments between the human genome and the chimpanzee or the macaque genomes (UCSC genome database http://genome.ucsc.edu/), we identified the substitutions occurring within the human lineage using maximum parsimony and assessed the number of substitutions within the human lineage that conserve ESE status (ESEs → ESEs) versus those that either create or erase an ESE. We compared the substitution patterns within the 20 nt flanking the 5′ and 3′ exon boundaries to the middle regions (20 nt) of lncRNA exons longer than 100 nt. The differences between internal regions and exon boundaries were tested using a χ^2^ test with 3 degrees of freedom.

We assessed potential selection on nucleotide composition within intergenic lncRNAs by implementing a test developed by Haddrill and Charlesworth (2008) derived from the McDonald and Kreitman test (McDonald and Kreitman 1991). At equilibrium, the following equality is expected:

$$\frac{{\text{Polymorphism}}_{\text{GC}\to \text{AT}}}{{\text{Divergence}}_{\text{GC}\to \text{AT}}}=\frac{{\text{polymorphism}}_{\text{AT}\to \text{GC}}}{{\text{divergence}}_{\text{AT}\to \text{GC}}}.$$

Any significant deviation could be attributed to the action of selection or other nonselective processes such as GC gene biased conversion (Haddrill and Charlesworth 2008).

To infer, using parsimony, the nucleotide composition of ancestral sequences, we used the genomic alignments of *D. melanogaster* with either *D. simulans* or *D. yakuba*. Sites whose ancestral state could not be inferred were discarded. We used these ancestral sequences to infer AT → GC and GC → AT directional substitution rates within lncRNA exons and introns (DuMont et al. 2009). For comparison we also computed rates for small introns (<86 nt) as a proxy for neutrally evolving sequences (Clemente and Vogl 2012).

## SUPPLEMENTAL MATERIAL

Supplemental material is available for this article.

## Supplementary Material

Supplemental Material
