# Supplementary material for: Unexpected selection to retain high GC content and splicing enhancers within exons of multiexonic lncRNA loci
Source: RNA. 2015 Mar;21(3):320–32. doi: 10.1261/rna.047324.114 (PMC4338330; doi:10.1261/rna.047324.114)
Supplement: Supplemental Material [file supp_21_3_320__index.html]

Unexpected selection to retain high GC content and splicing enhancers within exons of multiexonic lncRNA loci — Supplemental Material 

# Unexpected selection to retain high GC content and splicing enhancers within exons of multiexonic lncRNA loci

## Supplemental Material

**Files in this Data Supplement:**

- Supp Figure 1.pdf
- Supp Figure 2.pdf
- Supp Figure 3.pdf
- Supp Figure 4.pdf
- Supp Figure 6.pdf
- Supp Figure 5.pdf
- Supp Figure 7.xls
