# Supplementary material for: Unexpected selection to retain high GC content and splicing enhancers within exons of multiexonic lncRNA loci
Source: RNA. 2015 Mar;21(3):320–32. doi: 10.1261/rna.047324.114 (PMC4338330; doi:10.1261/rna.047324.114)

Supplementary Figure 1: Comparison of GC content between lncRNAs predicted to encode smORFs (< 100 aa, Bazzini et al. 2014) and the remaining lncRNAs in human. ns: not significant; *: P<0.05.

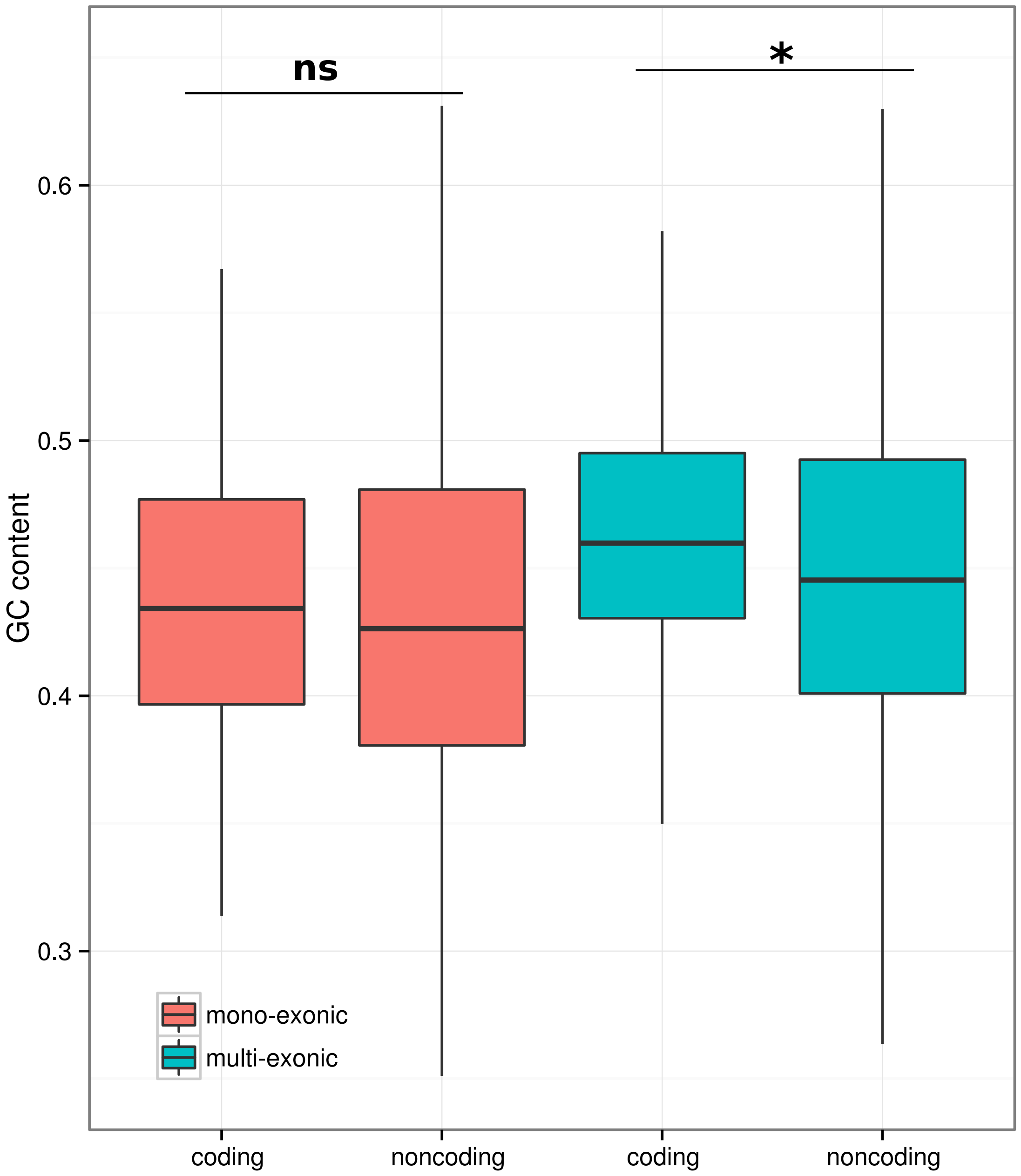

Supplement: Supplemental Material [file supp_047324.114_Supplementary_figure_1.pdf]
