# Supplementary material for: Unexpected selection to retain high GC content and splicing enhancers within exons of multiexonic lncRNA loci
Source: RNA. 2015 Mar;21(3):320–32. doi: 10.1261/rna.047324.114 (PMC4338330; doi:10.1261/rna.047324.114)

Supplementary Figure 2. GC content variation between lncRNAs exons depending upon gene model (mono-exonic, multi-exonic) and the presence of enhancers and TFBSs within the loci.

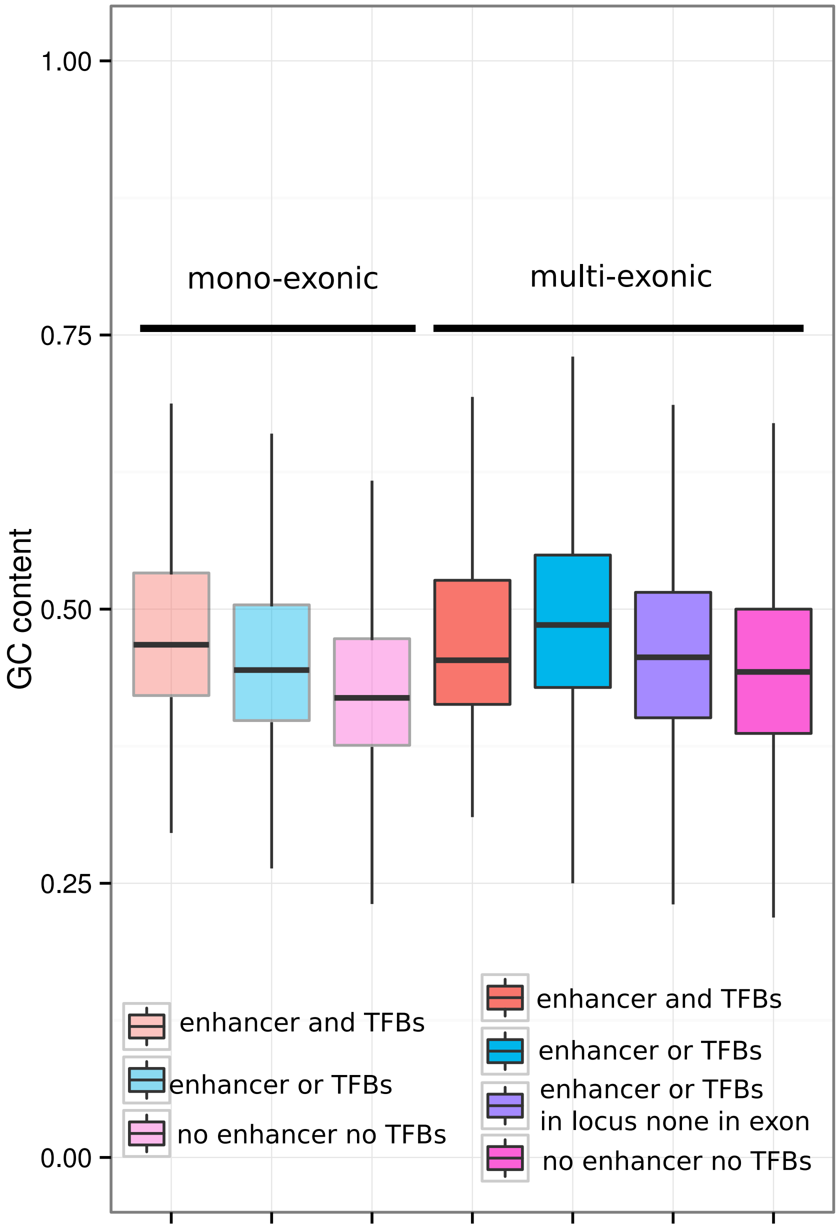

Supplement: Supplemental Material [file supp_047324.114_Supplementary_figure_2.pdf]
