# Supplementary material for: Unexpected selection to retain high GC content and splicing enhancers within exons of multiexonic lncRNA loci
Source: RNA. 2015 Mar;21(3):320–32. doi: 10.1261/rna.047324.114 (PMC4338330; doi:10.1261/rna.047324.114)

Supplementary figure 3: Comparison of GC content between exons with and without TFBSs within the same locus

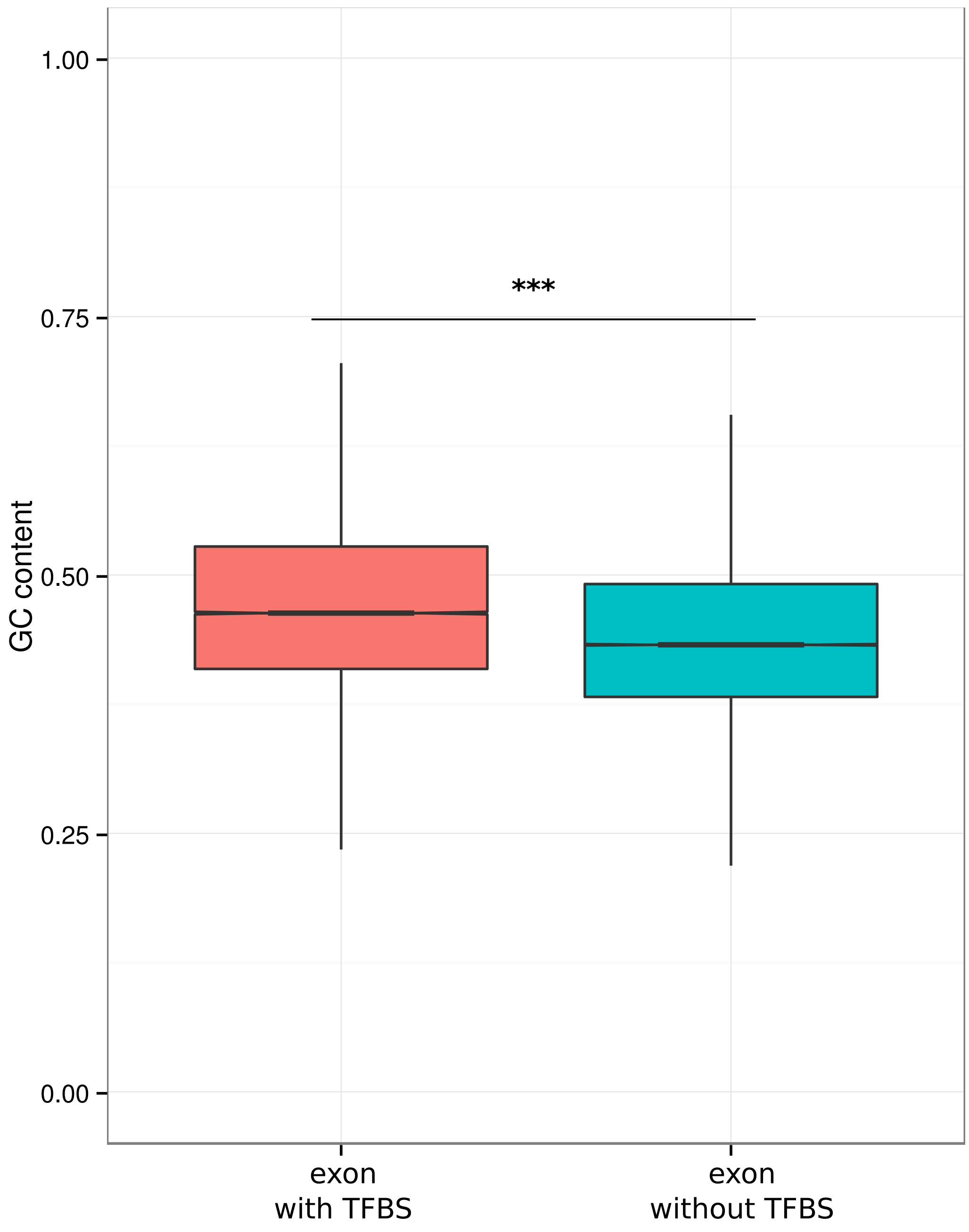

Supplement: Supplemental Material [file supp_047324.114_Supplementary_figure_3.pdf]
