# Supplementary material for: Unexpected selection to retain high GC content and splicing enhancers within exons of multiexonic lncRNA loci
Source: RNA. 2015 Mar;21(3):320–32. doi: 10.1261/rna.047324.114 (PMC4338330; doi:10.1261/rna.047324.114)

Supplementary Figure 4. Comparison of Maximum entropy scores (Yeo and Burge 2004) for protein-coding genes and lncRNA 5' and 3' splice sites classified according to the length of the adjacent intron in human.

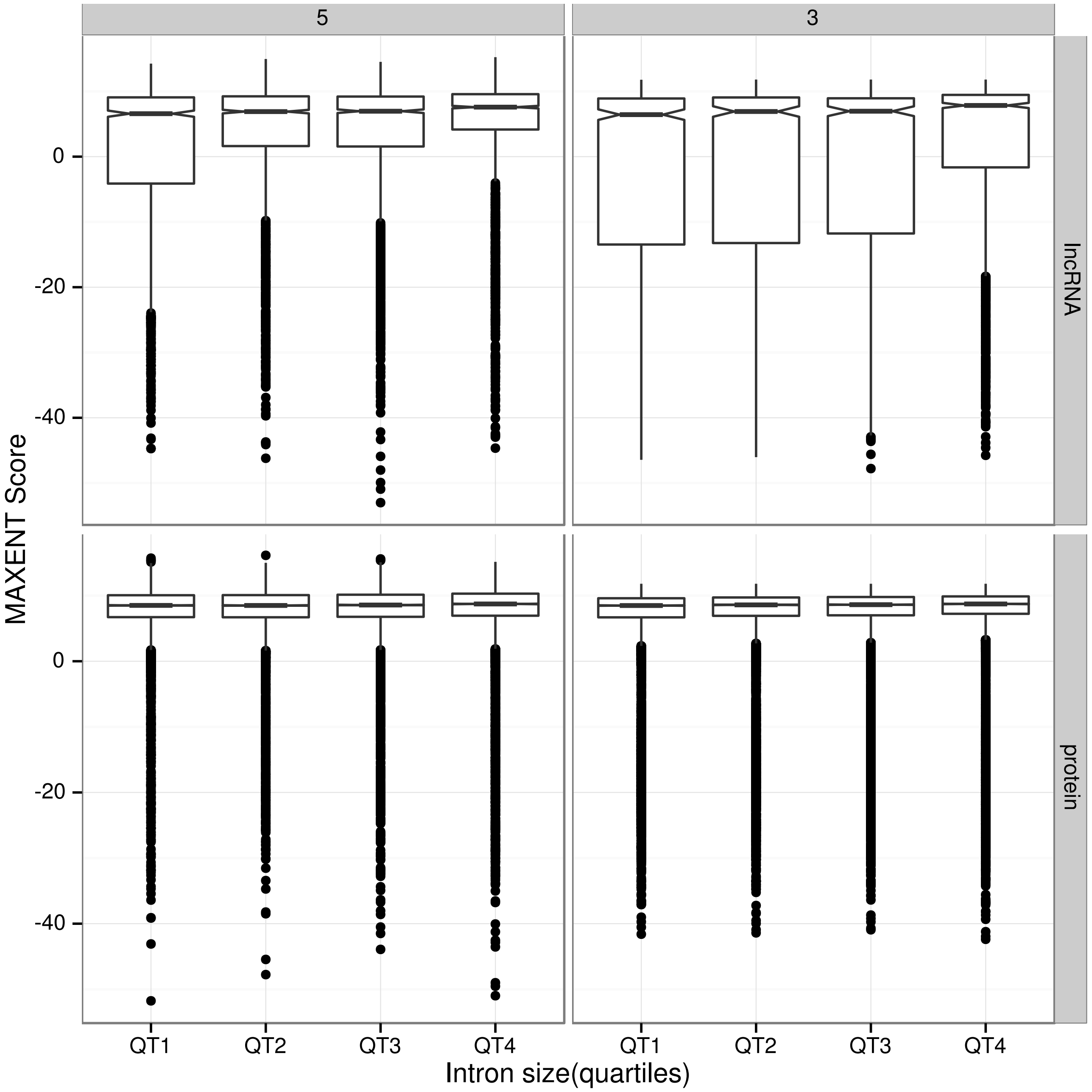

Supplement: Supplemental Material [file supp_047324.114_supplementary_figure_4.pdf]
