# Supplementary material for: Unexpected selection to retain high GC content and splicing enhancers within exons of multiexonic lncRNA loci
Source: RNA. 2015 Mar;21(3):320–32. doi: 10.1261/rna.047324.114 (PMC4338330; doi:10.1261/rna.047324.114)

Supplementary Figure 6. Comparison of the proportion of methylated CpG (mCpG) over the number of CpG at the 5' and 3' boundaries of intermediate protein coding (blue) and intergenic lncRNA (red) exons.

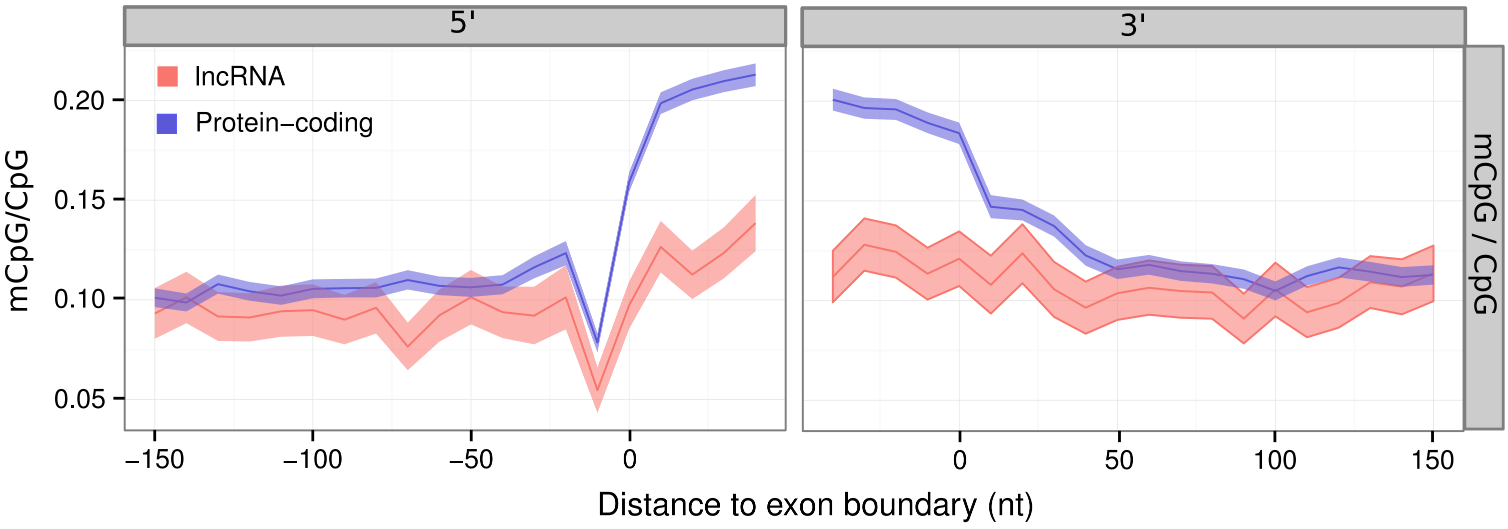

Supplement: Supplemental Material [file supp_047324.114_supplementary_figure_6.pdf]
