# Supplementary material for: Unexpected selection to retain high GC content and splicing enhancers within exons of multiexonic lncRNA loci
Source: RNA. 2015 Mar;21(3):320–32. doi: 10.1261/rna.047324.114 (PMC4338330; doi:10.1261/rna.047324.114)

Figure: Comparison of the observed (2743, red arrow) and the number of polymorphic sites within ESE sequences (predicted as per Fairbrother et al. 2002, Zhang and Chasin 2004, Goren et al. 2006) expected by simulation within human intergenic multi-exonic lncRNAs. The distribution of expected number of polymorphic sites within ESEs is based on 1000 randomizations of SNPs within multi-exonic lncRNA exons accounting for dinucleotide base composition.

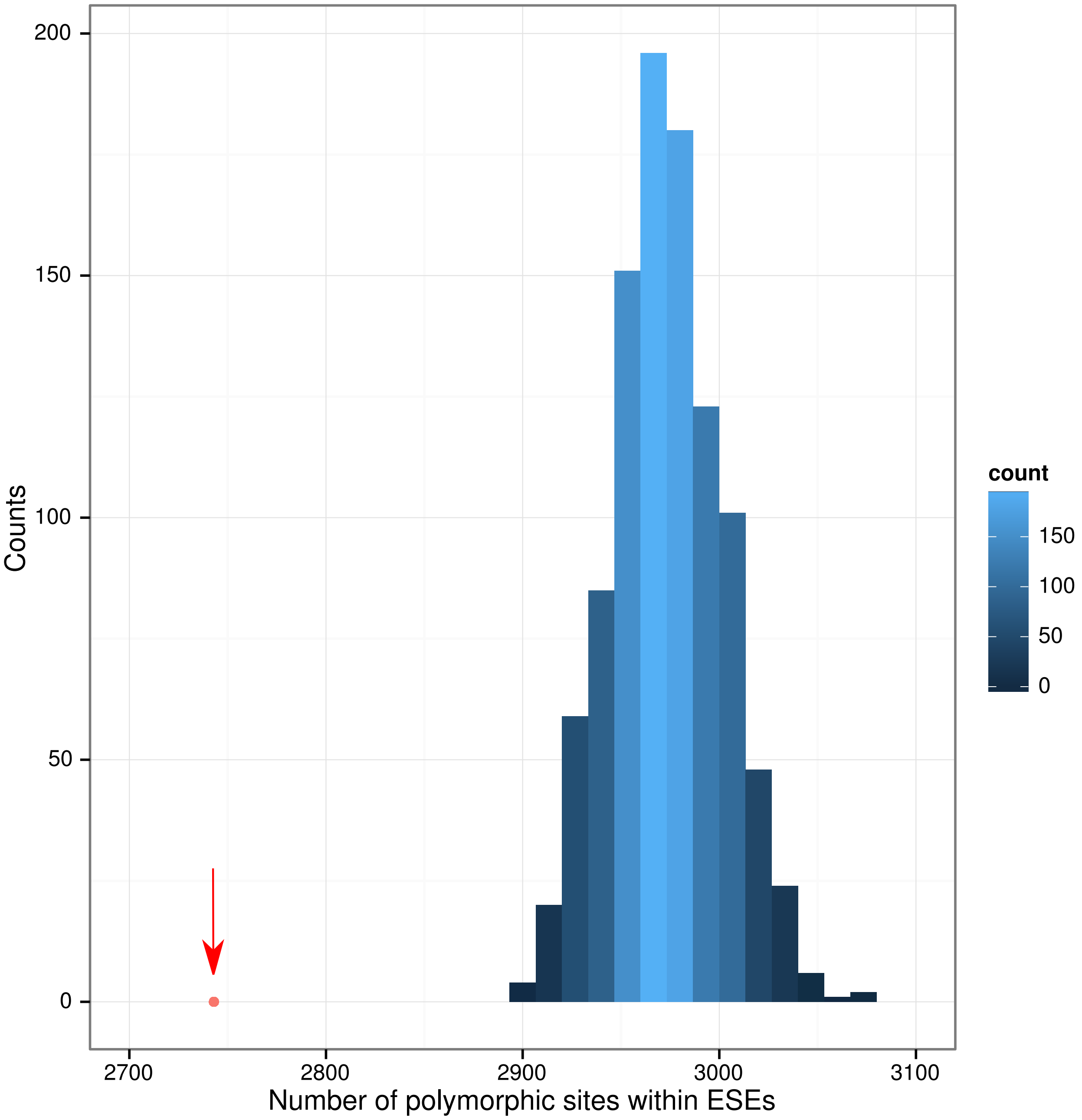

Supplement: Supplemental Material [file supp_047324.114_SuppFig5.pdf]
